# Management with Santorini’s Plexus Should Be Personalized during Prostatectomy

**DOI:** 10.3390/jpm12050769

**Published:** 2022-05-10

**Authors:** Jacek Wilamowski, Mateusz Wojtarowicz, Jan Adamowicz, Adam Golab, Michal Pozniak, Artur Leminski, Blazej Kuffel, Marcin Slojewski, Tomasz Drewa

**Affiliations:** 1 Department of Urology and Andrology, Collegium Medicum, Nicolaus Copernicus University, 85-089 Bydgoszcz, Poland; jacekwilamowski1986@gmail.com (J.W.); michalpozniak90@gmail.com (M.P.); blazej.kuffel@gmail.com (B.K.); t.drewa@wp.pl (T.D.); 2 Department of Urology and Urological Oncology, Pomeranian Medical University, 71-899 Szczecin, Poland; mateuszwojtarowicz@gmail.com (M.W.); adamgol@cyberia.pl (A.G.); artur.leminski@gmail.com (A.L.); mslojewski@gmail.com (M.S.)

**Keywords:** prostate cancer, prostatectomy, Santorini’s plexus

## Abstract

The aim of this study is to compare the results of laparoscopic prostatectomy in terms of management with Dorsal Venosus Complex (DVC)/Santorini’s plexus as it is still an open question in the field of urology. For this purpose, 457 patients after prostatectomy derived from two high volume centers were compared. In one center, patients underwent DVC ligation in all cases, whereas in the second center, this step was omitted. Subsequently, the histological and functional results were compared. Results showed that DVC management has an impact on blood loss and the duration of the surgery. In addition, omitting DVC ligation is demonstrated to reduce positive margin rate within the apex if the cancer was localized in this region. The continence and erectile function were similar in the 12-month follow up.

## 1. Introduction

The laparoscopic prostatectomy is an effective surgery and, despite dissemination of robotic technique, should not be abandoned yet, especially in high volume centers [1]. One of the advantages of laparoscopic prostatectomy due to its long presence in clinical practice is the availability of various studies’ results which allow for the personalization of the surgery’s technique. It is of utmost importance for a heterogonous disease, such as prostate cancer, for experts to optimize surgery steps in order to achieve the most favorable oncological and functional outcomes [2]. The management with DVC (Dorsal Venosus Complex)/Santorini’s plexus was always the field of recurrent discussion and controversy. As far as open surgery is concerned, it is an indispensable step, but the introduction of laparoscopy allowed experts to develop techniques omitting this procedure [3]. Although laparoscopic radical prostatectomy (LRP) is a well-established method, the influence of DVC management on surgery results still creates interest among the urological community.

## 2. Methods

### 2.1. Patients and General Characterization

This retrospective analysis included 457 patients who had clinically localized prostate cancer with indications for LRP, from two high volume centers. In center A (Department of Urology and Urologic Oncology PUM in Szczecin), all patients underwent DVC ligation whilst in center B (Department of Urology UNC in Bydgoszcz), this step was omitted in all cases. The DVC was ligated in a standard manner, after mobilization of prostate apex and before its dissection. Bleeding control without DVC ligation was only obtained using the Benique dilator to manually compress DVC towards symphysis. Additionally, the venous vessels were cut obliquely to facilitate their closing by insufflation pressure. Eligibility criteria in all enrolled patients included a cancer stage of <pT4 and no previous surgery or endoscopic prostate treatment (TUIP, TURP, or adenomectomy). We defined nerve sparing as preservation of NVB using a standard interfascial technique on at least one side. The demographic health status and perioperative variables were recorded: age, BMI, NYCHA scale, PSA, Gleason score, ISUP grade, operation time, estimated blood loss, specimen prostate weight, and surgical margin. Ninety days after surgery, complications were reported and graded using the Clavien–Dindo classification. The patients were followed for 12 months after the surgery, according to EAU-recommended schedule. None of the patients included in the study underwent adjuvant treatment. This study was monitored and approved by the local ethical committee (Bioethical Comity of Collegium Medicum, University of Nicolaus Copernicus in Torun), consent number KB102/2021.

### 2.2. Functional Evaluation

#### 2.2.1. Erectile Function

Erectile function and sexual performance were qualified and quantified 3, 6, and 12 months after surgery. All patients that declared themselves to be sexually active were screened using the IIEF-5 questionnaire before prostatectomy. In addition, sexual performance was defined as affirmative answers to the following: “do you have erections adequate for vaginal penetration?”— (Erection Hardness Score, EHS ≥ 3, Erection Hardness Score). There was no distinction between patients receiving PDE5 inhibitors to improve erection quality.

#### 2.2.2. Continence

The continence was estimated using the ICS-approved (International Continence Society) daily pad usage test in the following time periods: 3, 6, and 12 months after surgery. Patients defined as continent needed none or 1 pad daily. The degree of incontinence—mild, average, and severe—corresponded with daily pad demand: 1–2, 3, and ≥4 pads/24 h, respectively.

## 3. Statistical Methods

In order to compare the perioperative parameters in patients with supplied and unsupported Santorini plexuses, the analysis was performed with the Pearson χ^2^ test for nominal variables. For quantitative variables, the Mann–Whitney U test was used since the distribution of the variables differed significantly from the normal distribution. The level of significance was α = 0.05.

## 4. Results 

Table 1 presents the preoperative clinical characteristics of both groups. In terms of evaluated clinical factors that could influence decision making during prostatectomy or surgery outcomes, the groups were highly homogenic. Correspondingly, related clinical aspects that might independently affect potency or incontinence were also similar between groups (Table 2). Nerve sparing technique that is the most predictive factor for favorable outcome was applied in comparable number of cases in both centers [4]. As far as ASA score is concerned, the ASA I and ASA II grades predominated in examined population with ligated DVS whereas the number of ASA III patients was higher in the group with non-ligated DVS. The differences in ASA score were related to a large extent to higher number of IHD (ischemic heart disease) patients in second group.

Conducted comparison of the perioperative parameters showed the following differences (Table 3). Subgroup analysis confirmed that there was an evident correlation between operative time and DVC management. Omitting the plexus ligation shortened the operation time by an average of 42 min. In turn, however, the intraoperative blood loss was higher by 85mL if the DVC was not ligated (*p* < 0.001). Nevertheless, postoperative Hb count showed no differences between groups. Moreover, in both groups, patients were predominantly discharged after 2 days without noticeable difference in hospital stay.

## 5. Complications

All of the patients, complications occurred in 143 cases: 69 and 74 in groups with ligated and non-ligated DVC, respectively (Table 4). The applied approach to DVC management did not influence complication rate nor predispose to bleeding-related complication such as retropubic hematomas or prolongating hematuria. The reported complication profiles were typical for prostatectomy and did not differ between groups.

## 6. Histology

Histological analysis revealed that predominant cancer stage was pT2 in both groups (Table 5). The prostate cancer foci identified within apex were more common in the group with non-ligated DVC. The management with DVC did not influence overall incidences of positive surgical margins. Nevertheless, among patients with cancer localized in prostate apex, the positive margins in this region were significantly more frequently identified in the group with ligated DVC. Therefore, this interesting correlation may indicate that the apex resection plane is more accessible or controllable after skipping DVC ligation.

## 7. Continence

**Figure 1:**
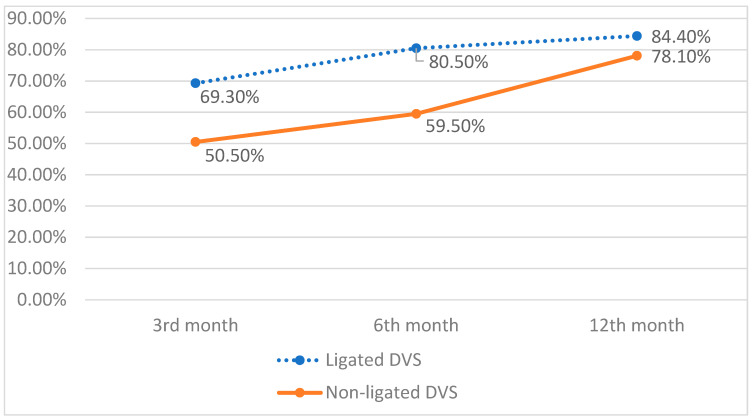
Continence recovery in 12 months follow up.

The preoperative clinical characteristics for both groups 1 and 2 are as follows: both groups had similar clinical factors that might independently affect potency, e.g., age, body mass index, and the presence of medical comorbidities (i.e., diabetes, coronary artery disease, etc.). The prevalence of the incontinence was more common in the group without DVC ligation 3 months after surgery (Figure 1). At that point in time, the satisfying continence rate was 50.50% and 69.30% in the non-ligated DVC group and the ligated DVC, respectively. Further follow-up demonstrated a statistically significant difference in continence rate between groups that was maintained 6 months post-surgery: 59.5% and 80.5%, respectively. Taking this result into consideration, the logistic regression analysis adjusted was additionally conducted to estimate the risk of incontinence occurrence. It turned out that DVC ligation reduced the risk of urinary incontinence by 64% (OR = 0.36) 6 months after surgery. Interestingly, at the end of the follow up 12 months after prostatectomy, there was no statistically significant difference in incontinence rates between groups. The conducted quality analysis of incontinent patients in both groups did not expose significant differences in incontinence grade during the follow up (Table 6). The only noticeable disproportion was found among severe incontinent patients 3 months after surgery.

## 8. Erectile Function

A total of 345 patients declared regular sexual activity in presurgical evaluation: 88.6% in the non-ligated DVC and 75.7% in the ligated DVC group.

**Figure 2:**
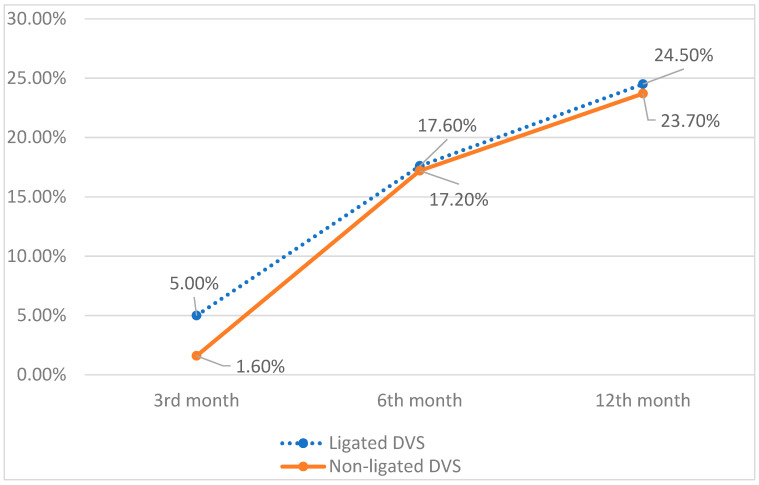
Erectile function recovery in 12 months follow up.

In the 3 months follow-up, the potency recovery was reported by 11 patients in both groups (8 ligated DVC; 3 non-ligated DVC) (Figure 2). In a further follow-up, 6 months after prostatectomy, 60 (28 ligated DVC and 32 non-ligated DVC) patients from both centers confirmed satisfactory erectile function. The final evaluation conducted after 12 months documented potency recovery in 83 men (39 ligated SVC; 44 non-ligated DVC). The noticed difference was insignificant, indicating that DVC management did not influence erectile function after prostatectomy.

## 9. Discussion

The most important outcome of prostatectomy is to obtain radical cancer resection [5]. In our study, the group without DVC ligation had a smaller number of positive margins in patients with cancer involving prostate apex. If the DVC is not ligated, the anatomical conditions for dissection of the prostate apex may be more favorable for the surgeon, in terms of both apex visibility and mobility [6]. These factors may, in turn, result in more efficient resection. The corresponding conclusions were reported by Guru et al. They demonstrated that robot-assisted laparoscopic radical prostatectomy (RALP) conducted without DVC ligation offered a significantly smaller number of positive margins within apex [7]. Analogously, Antonelli at al. indicated in randomized study that DVC ligation after prostate apex dissection decreased positive margin rate [8].

The major concern, in terms of DVC management, is perioperative blood loss and the risk of hemorrhage formation [9]. Despite slightly higher intraoperative blood loss, the Hb concertation after surgery was similar in both groups. This data is consistent with available research outcomes. However, in contrast to other studies, DVC ligation was not replaced with alternative maneuver aimed at preventing potential bleeding. Accordingly, Jarzemski et al. proposed to use tachosil sponge [10]. Porpiglia et al. demonstrated modification based on highly selective bipolar coagulation of DVC branches during dissection [11].

The patients that underwent prostatectomy without DVC ligation had a slightly higher ASA score. Worser physical status of this population may negatively impact functional results in the short term. ASA score is a strong predictive factor determining continence and erectile function after surgery. On the other hand, we did not notice a significant difference in side effect profile in a 30-day observation. One of the hypothetical explanations may be linked to shorter surgery duration if the DVC ligation was omitted. A shorter operation and related exposition on increased abdominal pressure, Trendelenburg position, etc. may compensate negative impact of the surgery on patient’s general health status and recovery.

Statistically, there were no differences in terms of incontinence rates 12 months after surgery in both groups. Only in the short-term follow-ups, 3 and 6 months after surgery, the incontinence occurred more frequently in patients without DVS ligation. This observation implied that DVC ligation might reduce the neuropraxia within neuronal network over prostate apex. The DVC ligation provides “per se” partial stabilization of frontal apex dissection plane by anchoring the DVC to the pubic symphysis [12]. This may help to reduce tension on nerve fibers running and branching in this area. Nerve fibers originating from the pelvic plexus and supplying sphincter complex and corpus cavernosa are generally unmyelinated and hence particularly prone to tension or ischemic related neuropraxia [13,14]. Hoshi et al. demonstrated superior continence results in group with spared DVC over standard management [15]. Similar to our results, the most evident difference was observed in the 3- and 6-month follow ups after surgery. The concept of DVC suspension was then proven in robot-assisted laparoscopic radical prostatectomy [16]. The robotic surgery offers excellent access to prostate apex and tools maneuverability range that allowed to develop and then evaluate multiple approaches to DVC management including endoscopic stapling, cut and suture ligation, and suture ligation with suspension [17]. It was demonstrated that the best results guaranteed ligation with suspension and the difference was the most striking between compared groups in a short follow-up.

During surgery with DVC ligation, a vesicourethral anastomosis was performed only with the use of single sutures. Whereas in second center, the continuous suture on anterior anastomosis wall was applied. Despite this difference, considering available research, the type of anastomosis single vs. continuous does not impact prostatectomy outcomes [18].

Similarly, erectile function is always compromised after prostatectomy [19]. In general, the erectile function recovery is worse in comparison to continence due to lack of compensatory mechanism. The research data analyzing influence of DVC management on erectile recovery is inconclusive [20,21,22,23]. The potential explanation may be related to individually variable pathways of cavernous nerve and its supplying branches running often in the pudendal nerve [24]. Vast bundles of cavernous nerve concentrate in the prostate apex region and their damage during resection is impossible to avoid. According to available data, DVC ligation may improve erectile function in a short follow-up, although we did not notice such a correlation [25]. Based on reported high volume center experience, surgeon’s skill and degree of nerve sparing, patient age and preoperative potency, are the greatest predictors of postoperative potency outcomes. Paradoxically, DVC ligation may improve erection after surgery due to the enhancement of the veno-occlusive mechanism [26].

## 10. Limitation and Conclusions

The limitation of the study includes its two-center design. In terms of prostatectomy, which is a multistep procedure and all of surgery stages may be customized by a surgeon, there is a risk of center bias. Therefore, in this study, two centers with constituted experience in a particular technique were included. Moreover, comparing functional results after prostatectomy is difficult in research without very strict randomization because, in fact, there are no standardized nor repetitive protocols. The unification of recommendations regarding uro-rehabilitation and postprostatectomy care aimed at improving erectile function should be addressed by working groups of urological scientific community.

Personalization of the prostatectomy is a natural development pathway of modern urology. The rich study data enable surgeons to obtain plenty of information that could be used to adopt surgery technique to a patient’s needs. For instance, if prostate MRI imaging before surgery exposes cancer in prostate apex, it may be reasonable to consider an adequate DVC management which aims to reduce the risk of positive margin. Flexible modification of surgery method, based on presurgical planning, allows surgeons to avoid routine and to provide the best results for patients. The summarized study results demonstrate that a different approach to DVC management marginally influenced prostatectomy outcomes. Therefore, the selection of a specific technique should be left to the surgeon’s decision.

## Figures and Tables

**Table 1:** Preoperative characteristic of patients included in the study.

	All Patients *n* = 415 (%)	Ligated DVC *n* = 205 (%)	Non-Ligated DVC *n* = 210 (%)	*p*-Value
ISUP/Gleason grade group,				0.439
ISUP 1/Gleason 6	246 (59.3)	120 (58.5)	126 (60)	
ISUP 2/Gleason 7 (3 + 4)	82 (19.8)	42 (20.5)	40 (19)	
ISUP 3/Gleason 7 (4 + 3)	40 (9.6)	19 (9.3)	21 (10)	
ISUP 4/Gleason 8	40 (9.6)	22 (10.7)	18 (8.6)	
ISUP 5/Gleason 9 i 10	7 (1.7)	2 (1)	5 (2.4)	
PSA (ng)				0.513
min. max.	1.5–94	1.65–94	1.5–90	
median	12.03	12.12	11.94	
≤10	254 (61.2)	123 (60)	131 (62.4)	
10, 1–20	112 (27)	54 (26.3)	58 (27.6)	
>20	49 (11.8)	28 (13.7)	21 (10)	
Cancer stage				0.439
cT1a-b	0 (0)	0 (0)	0 (0)	
cT1c-T2	377 (90.8)	189 (92.2)	188 (89.5)	
cT3	38 (9.2))	16 (7.8)	22 (10.5)	
cT4	0 (0)	0 (0)	0 (0)	
Prostate volume (mL)				0.568
min. max.	15–160 mL	15–100 mL	15–160 mL	
median	43.3 mL	41.95 mL	44.68 ml	
≤30	113 (27.2)	60 (29.3)	53 (25.2)	
30, 1–50	208 (50.1)	102 (49.8)	106 (50.5)	
>50	94 (22.7)	43 (21)	51 (24.3)	
D’Amico Classification				0.412
low risk	125 (30.1)	48 (23.4)	77 (36.7)	
intermediate risk	165 (39.8)	87 (42.4)	78 (37.1)	
high risk	125 (30.1)	70 (34.1)	55 (26.2)	

**Table 2:** Characteristics of comorbidities.

	All Patients *n* = 415 (%)	Ligated DVC *n* = 205 (%)	Non-Ligated DVC *n* = 210 (%)	*p*-Value
Age				
min. max.	45–84	45–76	45–84	
median	64.5	64.08	64.91	0.179
BMI				
min. max.	17.3–39.18	19.62–38.53	17.3–39.18	
median	27.86	27.75	27.97	0.530
Arterial hypertension	229 (55.2)	108 (52.7)	121 (57.6)	0.362
Ischemic heart disease	72 (17.3)	22 (10.7)	48 (22.9)	0.002
Diabetes	53 (12.8)	22 (10.7)	31 (14.8)	0.279
Asthma	15 (3.6)	4 (2)	11 (5.2)	0.126
Atrial fibrillation	14 (3.4)	4 (2)	10 (4.8)	0.189
ASA I	39 (9.4)	37 (18)	3 (1.4)	<0.001
ASA II	279 (67.2)	163 (79.5)	116 (55.2)	
ASA III	97 (23.4)	6 (2.9)	91 (43.3)	
ASA IV	0 (0)	0 (0)	0 (0)	

**Table 3:** Perioperative parameters.

Parameter	All patients *n* = 415 (%)	Ligated DVC *n* = 205 (%)	Non-Ligated DVC *n* = 210 (%)	*p*-Value
Surgery duration (min.)				<0.001
min.max.	50–230 min	80–230 min	50–185 min	
median	119 min	140 min	98 min	
Hospital stay (days):				>0.05
min.max.	1–15	1–7	1–15	
median	2.7	2.7	2.7	
Intraoperative blood loss (mL):				<0.001
min.max.	0–1800 mL	0–1000 mL	10–1800 mL	
median	266 mL	223 mL	30	
≤100 mL	86 (20.7)	47 (22.9)	39 (18.6)	
101–200 mL	140 (33.7)	82 (40.0)	58 (27.6)	
201–500 mL	144 (34.7)	64 (31.2)	80 (38.1)	
>500 ml	45 (10.9)	12 (5.9)	33 (15.7)	
NVB sparing:				>0.05
Bilateral	275 (66.2)	133 (64.9)	142 (67.6)	
Unilateral	31 (7.5)	14 (6.8)	17 (8.1)	
Abandon	109 (26.3)	58 (28.3)	51 (24.3)	
Hb decrease (g/dL):				0.921
min.max.	0.2–7.9 g/dL	0.6–6.4 g/dL	0.2–7.9 g/dL	
median	3.17 g/dL	3.15 g/dL	3.20 g/dL	
Drain leak (mL):				0.155
min.max.	0–2400 mL	0–2400 mL	0–1360	
median	237 mL	298 mL	177 mL	
≤100 mL	164 (39.5)	79 (38.5)	85 (40.5)	
101–200 mL	108 (26.0)	38 (18.5)	65 (31.0)	
201–500 mL	101 (24.5)	54 (26.3)	44 (21.0)	
501–1000 mL	21 (5.0)	11 (5.4)	8 (3.8)	
>1000 ml	21 (5.0)	16 (7.8)	2 (1.0)	

**Table 4:** Ninety days complications according to the Clavien–Dindo classification.

Clavien–Dindo Grade	Ligated DVC	Non-Ligated DVC	*p*-Value
Grade I (67)	38	29	0.340
Lymphocele	25	18	0.444
Anastomosis Leakage	5	3	0.499
Wound infection	0	6	0.030
Limb lymphedema	3	0	0.120
Hematoma	3	1	0.367
Obturator nerve injury	2	1	0.620
Grade II (73)	31	42	0.264
UTI	12	17	0.869
Blood transfusion	10	14	0.889
Intraoperative rectal injury	2	4	
Hematuria	5	5	>0.999
Thrombosis	1	1	0.543
Ileus	1	1	>0.999
Grade IIIa (44)	16	29	0.113
Percutaneous drainage (lymphocele. abscess. hematoma)	14	24	0.278
Nephrostomy	1	2	0.499
Suprapubic cystostomy (Anastomosis Leakage)	1	3	0.623
Grade IIIb 17	13	4	0.042
laparotomy (rectal injury)	2	2	>0.999
Laparotomy	4	0	0.059
Laparotomy (anastomosis leak)	1	0	0.494
fenestration of lymphocele	1	0	0.494
Anastomosis stricture	3	0	0.120
Orchidectomy	2	1	0.256
Postoperative hernia surgery	0	1	>0.999
Grade IV. V (6)	2	4	0.685
Urosepsis	1	2	>0.999
Pulmonary embolism	1	1	>0.999
Myocardial infarction	0	1	>0.999

**Table 5:** Histological analysis.

	All Patients *n* = 415 (%)	Ligated DVC *n* = 205 (%)	Non-ligated DVC *n* = 210 (%)	*p*-Value
Cancer stage				
pT2	276 (66.5)	141 (68.8)	135 (64.2)	0.387
pT3	139 (33.5)	64 (31.2)	75 (35.8)	0.387
pT3a	79 (19.0)	41 (20.0)	38 (18.2)	0.712
pT3b	60 (14.5)	23 (11.2)	37 (17.6)	0.087
pT4	0 (0)	0 (0)	0 (0)	
Positive surgical margin				
Overall	139 (33.5)	78 (38)	61 (29)	0.052
Right	35 (8.4)	22 (10.7)	13 (6.2)	0.137
Left	34 (8.2)	16 (7.8)	18 (8.6)	0.916
Bilateral	22 (5.3)	11 (5.4)	11 (5.2)	1.000
Apex	58 (14.0)	34 (16.6)	24 (11.4)	0.145
Positive surgical margin	139 (33.5)	78 (38.0)	61 (29)	0.052
Positive surgical margin—apex (all patients)	58 (18.9)	34 (26.2)	24 (13.6)	0.145
Cancer identified in apex	307 (74.0	130 (63.4)	177(84.3)	<0.001
Positive surgical margin—apex (cancer localized in apex)	58	34 (26.2)	24 (13.6)	0.005
ISUP/Gleason grade:				
ISUP 1/Gleason ≤ 6	134 (32.3)	57 (27.8)	77 (36.7)	0.081
ISUP 2/Gleason 7 (3 + 4)	178 (42.9)	94 (45.9)	84 (40.0)	0.259
ISUP 3/Gleason 7 (4 + 3)	52 (12.5)	31 (15.1)	21 (10.0)	0.342
ISUP 4/Gleason 8	36 (8.7)	18 (8.8)	18 (8.5)	0.945
ISUP 5/Gleason 9 I 10	15 (3.6)	5 (2.4)	10 (4.8)	0.201

**Table 6:** Evaluation of incontinence grade in 12 months follow up.

Incontinence Grade ICS Scale	Ligated DVC	Non-Ligated DVC	
	3 months Number of patients		
Mild	32	29	*p* > 0.05
Average	19	34	
Severe	12	41	*p* = 0.004
	6 months		
Mild	23	43	*p* > 0.05
Average	11	28	
Severe	6	14	
	12 months		
Mild	32	46	*p* > 0.05
Average	21	26	
Severe	3	7	
